# Is occupational exposure to solvents associated with an increased risk for developing systemic scleroderma?

**DOI:** 10.1186/1745-6673-1-15

**Published:** 2006-07-03

**Authors:** Birgitta Kütting, Wolfgang Uter, Hans Drexler

**Affiliations:** 1 Institute and outpatient clinic of occupational, social and environmental medicine (head: Prof. Dr. H. Drexler), University of Erlangen-Nuremberg, Schillerstr. 25 + 29, D-91054 Erlangen, Germany; 2 Dept. of Medical Informatics, Biometry and Epidemiology (head: Prof. Dr. O. Gefeller), University of Erlangen-Nuremberg, Waldstr. 6, D-91054 Erlangen, Germany

## Abstract

**Background:**

Our study was aimed to investigate in a German collective if there are any hints for an increased occupational or environmental risk to develop systemic sclerosis, especially, focussing on work-related exposure to solvents. Moreover, we tried to evaluate the feasibility of a sampling method addressing support groups.

**Methods:**

A standardised questionnaire was published in two journals subscribed by members of two different support groups and all members were asked to complete the questionnaire and to return it anonymously. The subjects were not informed on the scientific hypotheses, nor did they know who of them belonged to the case group (scleroderma) or to the control group (multiple sclerosis).

**Results:**

175 questionnaires could be included in the statistical analysis. As expected, a female predominance was in our collective. In the male subpopulation, the occupational exposure to solvents was higher in the case group than in the control-group (70% versus 45.8%).

Based only on the male subgroup, a tendency for an association between occupational exposure to solvents and the risk to develop systemic sclerosis was found.

**Conclusion:**

According to our experience in this case-control-study exposure misclassification, qualitative or quantitative, was an eminent problem. Within such a setting, it is generally very difficult to establish an exact dose-response relationship due to incomplete, imprecise or missing data concerning duration of exposure, frequency of use and kind of solvent. Additionally, a well-known problem in studies based on self-reported questionnaires is the so-called volunteer bias. Unfortunately, but similar to other studies assessing epidemiologic factors in such a rare disease, our study was of limited power, especially in the subgroups defined by gender.

## Background

Systemic sclerosis is a rare multisystem disease with a reported incidence of 2 to 12 cases per million people per year [1]. The disease is characterized by microvascular alteration and massive deposition of collagen affecting connective tissue in many parts of the body, especially skin, oesophagus, lungs, gastrointestinal tract, kidney, heart and other internal organs [2]. The aetiology of systemic sclerosis still remains unclear. Data of epidemiological studies suggest a complex interaction of genetic, hormonal and environmental factors in the pathogenesis of fibrotic changes in scleroderma [3]. Twin studies have shown that the occurrence of systemic sclerosis in monozygotic twins is rare suggesting a lesser role for genetics [4,5]. In addition, familial clustering has not been found [6]. Environmental factors may play a more important role in the pathogenesis of systemic scleroderma. For instance, spouses of affected individuals have an increased occurrence of autoantibodies, suggesting a shared environmental exposure [7]. Ethnic susceptibility has been demonstrated in Thai patients, who have a higher incidence of anticentromer antibodies, and who are more likely to have diffuse disease compared with Caucasians and Australians [8]. Although hormones are supposed to play a role in the aetiology of the disease in women, a case control study failed to indicate an association between systemic sclerosis and contraceptive use, earlier age of menarche, or ever being pregnant [9].

A number of drugs have been reported to be associated with the development of scleroderma-like disease, including bleomycin, pentazocine, cocaine, appetite suppressants and D-penicillamine [10].

Up to now, several work-related factors such as exposure to silica dust, epoxy resins, organic solvents or work-related hand-arm vibrations had been identified as important risk factors for developing scleroderma [1,3]. In fact, almost 50 years ago Reinl [11] was the first to point out the association between systemic sclerosis and exposure to solvents.

Since then several case reports and series [12-17] as well as epidemiologic studies [18-21] have found systemic sclerosis to be positively associated with exposure to solvents. Supportive of a causal association, a biologically plausible link between organic solvent exposure and systemic sclerosis had been hypothesized: Organic solvents and their reactive metabolites are supposed to bind covalently to protein molecules such as topoisomerase I (Scl-70) and may stimulate an autoimmune response [6].

Our study was aimed to investigate the relationship between certain environmental or occupational exposures and systemic scleroderma in a German population, hereby focussing on the exposure to solvents.

At the same time, we critically evaluated the chosen approach of accessing the study population.

## Methods

A standardized questionnaire was published in two journals subscribed by members of two different self-help associations and all members were asked to complete the questionnaire and to return it anonymously to us. One of the journals was the official organ of a self-help group for patients suffering from scleroderma (cases), whereas the other journal was subscribed by members of a multiple sclerosis support group (control-group).

As control group, patients suffering from multiple sclerosis were selected, because similar to systemic sclerosis, the aetiology is almost unknown and hence the administration of a questionnaire addressing a variety of potential risk factors to this group appeared credible. Moreover, in both diseases female patients predominate, offering the chance of some degree of matching for this variable. The subjects were not informed on our working hypotheses, nor did they know who of them belonged to the case group or to the control group. The questionnaire consisted of 24 items, including some irrelevant questions, to camouflage the exposures of interest. The first part of the questionnaire consisted of questions concerning general anamnestic data such as sex, date of birth, date of diagnosis and the parents' country of origin. The second part of the questionnaire was focussed on professional activities and exposure to harmful substances such as solvents, metallic fumes, dust and hazardous working conditions such as hand-arm vibration. If the questions were answered in the affirmative we asked for the duration of exposure during the working-life, frequency of exposure and average time of exposure. The last part of the questionnaire asked for the intake of drugs inducing scleroderma-like disease, silicone gel-filled implants, infections, private exposure to solvents and diet. The number of copies of both journals was 2000 each. The scleroderma support group was supposed to have approximately 1500 members; of these nearly 900 were known to suffer from a systemic form of this disease. Only persons suffering from systemic sclerosis were asked to complete the questionnaire. Potential confounders such as age, sex and race were considered, in that analysis of the association between exposures and case status were adjusted for age (as median dichotomized categorical variable) and stratified for sex in logistic regression analysis, and only Caucasians were included in the analysis.

## Sample

175 questionnaires could be included in the statistical analysis. 109 questionnaires were returned by members of the scleroderma self-help group, leading to a response rate of 7.3%, based on the total number of members. Related to the number of persons suffering from a systemic form of the disease and these subjects were only asked to complete our questionnaire, the response rate was 12.1%. In contrast, we received only 66 questionnaires from persons suffering from multiple sclerosis. As expected, we had a female predominance in our collective (especially in the case group). In the case group, 99 women and 10 men completed and returned the questionnaire, whereas in the control group data of 42 women and 24 men could be included in the analysis (p < 0.0001, Fisher's exact test).

## Results

The mean age of persons suffering from scleroderma was 57 years (median 59 years, range 20 to 79 years). The average age of the female study population was 58 years, whereas the men were 49 years old. The mean age of persons suffering from multiple sclerosis was 45 years (median 43 years, minimum 26 years, and maximum 75 years), i. e, somewhat younger. The women of the control group were on average 43 years old, the men 47 years. All subjects were Caucasian.

The female study population had been aware of the diagnosis of systemic sclerosis for 10 months, averaged (minimum 1 month, maximum 31 months, standard error 7.6), whereas the male study population reported on knowing their diagnosis for 8 months at average (minimum: 1 month, maximum 23 months, standard error 6.9). In the control group the duration of diagnosis was indicated with almost 1 year on average (minimum 1 month, maximum 36 month, deviation 8.8) in the female subgroup, the averaged indicated duration of diagnosis had been almost ten month in the male subgroup. Therefore, the mean duration of diagnosis was stated with 10 months in the study population versus 11 months in the control group. The higher frequency of occupational and private exposure to solvents in the control-group goes along with a higher percentage of men (33% men in the control group versus 9.2% in the study group). In the control-group, 33.3% (n = 22) reported work-related exposure to solvents, whereas 56.1% (n = 37) indicated an exposure to solvents due to private activities. However, in the female subpopulation, the occupational exposure to solvents was higher in the control-group (26.2%) compared to the case group (12.1%). In the male subpopulation the occupational exposure to solvents was higher in the case group than in the control-group (70% versus 45.8%). The indicated frequency of occupational exposure to solvents was the highest in the male study population, 4 study participants indicated occupational exposures to solvents more than four times a week (table 1). The female study population reported on 2 years and 1 month averaged occupational exposure to solvents, whereas the male study population indicated a mean of almost 12 and a half years (versus female control group: 3.2 years, male control group 4.3 years). Exposure to solvents due to private activities such as renovation was reported in 40.4% of all cases in the study group compared to 56.1%, in the control-group. 10% of all cases of scleroderma (7 women, 3 men) indicated as well occupational as private exposure to solvents, whereas 22.7% reported this in the control group (5 women, 10 men) (table 2).

**Table 1:** Frequency of occupational exposure to solvents

**Frequency**	**Cases female (n/%)**	**Controls female (n/%)**	**Cases male (n/%)**	**Controls male (n/%)**
missing data	0	1	0	1
	0%	2.4%	0%	4.2%
never	85	31	3	13
	85.9%	73.8%	30%	54.2%
2x/6 months	0	0	0	2
	0%	0%	0%	8.3%
2x/3 months	0	0	0	1
	0%	0%	0%	4.2%
1x/month	2	0	0	2
	2%	0%	0%	8.3%
1x/week	0	0	2	1
	0%	0%	20%	4.2%
2x/week	0	1	1	0
	0%	2,4	10%	0%
3x/week	1	0	0	2
	1%	0%	0%	8.3%
4x/week	0	0	1	0
	0%	0%	10%	0%
5x/week	6	5	2	1
	6.1%	11,9%	20%	4.2%
6x/week	3	2	1	0
	3%	4,8%	10%	0%
daily	0	2	0	1
	0%	4,8%	0%	4.2%

**Table 2:** Possible risk for developing scleroderma related on known occupational risk factors, quantified with the odds ratio (OR) as derived from three separate logistic regression analyses, adjusted for age (<than overall median versus> = overall median).

	**Females (n = 141)**				**Males (n = 34)**			
		
	**Cases (n = 99) n/%**	**Controls (n = 42) n/%**	**OR [95% CI]**	**GOF**	**Cases (n = 10) n**	**Controls (n = 24) n**	**OR [95% CI]**	**GOF**
Exposure to solvents:				0.5768				0.8722
None	53 (53.5%)	17 (40.5%)	1.00 (reference)		2	6	1.00 (reference)	
Private only	31 (31.3%)	13 (31.0%)	1.047 (0.397–2.815)		1	7	0.427 (0.017–5.618)	
Occupational only	5 (5.1%)	4 (9.5%)	0.480 (0.086–2.644)		3	2	4.794 (0.459–69.901)	
Both	6 (6.1%)^#^	5 (11.9%)^#^	0.439 (0.089–2.094)		4	9	0.427 (0.017–5.618)	

Occupational hand-arm vibration (any vs. none)	5 (5.1%)	1 (2.4%)	2.961 (0.358–62.605)	0.8970	4	6	2.050 (0.378–11.312)	0.5780

Occupational exposure to metallic fumes or dust (any vs. none)	8 (8.1%)	4 (9.2%)	1.445 (0.366–6.309)	0.8398	7	10	3.319 (0.708–19.193)	0.8832

GOF: goodness of fit (p value of Hosmer and Lemeshow test)

^# ^remainder: missing

Logistic regression analysis was performed, adjusting for age (median dichotomized) and stratified for gender, to derive gender-specific risk estimates. Due to the limited size of the sample, exposure to solvents, occupational hand-arm vibration and occupational exposure to metallic dusts and fumes were considered in three different logistic regression models. The results indicate that exposure to solvents – whether occupationally related or private – is generally not a risk factor for systemic scleroderma; only in the subgroup of males a trend for increased risk can be noted. Conversely, occupational hand-arm vibration is associated with an increased risk, much lesser so also occupational exposure to metallic fumes. Model fit, as assessed with the Hosmer and Lemeshow test, was good to excellent in all cases (Tab. 2).

## Discussion

In our study population a general positive association between exposure to solvents and systemic scleroderma could not be confirmed. Only in the male subgroup a weak [non-significant] association between occupational exposure to solvents and the risk of having systemic sclerosis was observed. Unfortunately, but similar to other studies in this field, our study was of limited power, especially in the subgroups defined by gender.

A meta-analysis published three years ago by Aryal et al. [18] confirmed a significant positive association between exposure to solvents and systemic sclerosis. This meta-analysis was based on 7 case-control-studies and one cohort study, the number of cases varying between 21 and 274 cases. However, due to limited presentation of subgroup data, the authors were unable to perform separate meta-analyses for male and female subjects. It was supposed that most of the studies included had a greater number of female cases, reflecting the female predominance of this disease. Among the studies included in the analysis, all but one [22] showed an association between systemic sclerosis and solvents. The so-called publication bias, a phenomenon of selective submission or acceptance of research based on the attainment of statistically significant positive correlation, is probably responsible for the preponderance of studies reporting an association between exposure to solvents and the risk to develop systemic sclerosis. However, the only study with the missing association was performed in a strictly male study population (number of cases n = 56). Recently, controversial to the findings of Silman and Jones [22], a tendency for an association between occupational exposure to solvents and scleroderma had been observed only in male subjects [21]. The data of Bovenzi are very well in line with our findings, even if both male subpopulations, due to the female predominance of this disease, were very small with 9 [21] and 10 subjects, respectively.

Apart from publication bias, different types of bias may limit the findings of such a case-control-study. Age, gender and race are considered to be potential confounders of systemic sclerosis, but these confounders have not always been taken into account. Furthermore, due to the rarity of the disease; it might be difficult – due to logistic reasons – to include a large number of subjects. Therefore, most studies are based on a small number of subjects, with correspondingly imprecise effect estimates. Usually it might be extremely difficult to recruit the control-group and to convince the control subjects to take part in the trial. Additionally, there might be a huge difference between cases and controls in the attitude of answering the questionnaire. To lessen or avoid this bias we decided to include only patients (as cases and controls), who, from their perspective, might all have acted as case group, but not informing the participants who of them belonged to the case-group and who became part of the control-group. However, as some of the evidence concerning an aetiological role of solvents may have transpired to the systemic sclerosis self-help association's members, but presumably not to the MS group, we cannot rule out recall bias completely.

A well-known problem in studies based on self-reported questionnaires is the so-called volunteer bias, a special variant of selection bias. This bias implies that (i) the overall response rate strongly depends how far subjects feel addressed by the questions and suppose these questions to be relevant to reflect their personal situation and (ii) those subjects who consider (some of) the exposures of interest as relevant for their illness may selectively respond. Therefore, we tried to develop a diversified questionnaire related to different topics. All work-related questions were embedded and mixed up with questions concerning the past medical history, life style and nutrition in order to camouflage our special interest in occupational exposure to solvents. Assuming that "housewives", unemployed or retired subjects probably would not see the necessity to answer work-related questions; we did our best to mask our hypothesis as much as possible.

Although we were unable to employ the clear inclusion criteria and a face-to-face examination for case ascertainment often used in a case control study with the study design chosen, and hence some degree of misclassification of the disease status may have occurred, we assume that registration in a self-help group is a valid surrogate of a physician based diagnosis of either disease. Furthermore, exposure misclassification, qualitative or quantitative, is often a major problem in case-control studies. Within such a setting, it is generally very difficult to establish an exact dose-response relationship due to incomplete, imprecise or missing data concerning duration of exposure, frequency of use and kind of solvent. In most studies exposure assessment usually relies on subjective statement. Only in three studies [19,20,23] the authors tried to perform an objective method of exposure assessment in their case-control study. In the study of Garabrant et al. [20] an expert in exposure assessment was asked to review and classify the exposure histories and to assign probability and plausibility of exposures.

Plausibility of our data was proofed by several questions related to the same item and giving concordant results, e.g. the question asking for occupational exposure to solvents with the possibility to answer by yes or nor and then the question related to the frequency of occupational exposure to solvents offering different possibilities to answer from never to daily on a scale. All subjects indicating none occupational exposure to solvents gave later the answer that they never used solvents at work.

## Conclusion

In conclusion, further epidemiological studies to evaluate the association of work-related exposure to solvents and systemic scleroderma in male subjects are deemed necessary, as our study was able to contribute not more than weak evidence in this matter. In these studies, strategies for better ascertainment of exposure history to solvents should probably be employed.
